# Conformational entropy of a polymer chain grafted to rough surfaces

**DOI:** 10.1007/s00894-012-1546-5

**Published:** 2012-08-24

**Authors:** Waldemar Nowicki, Grażyna Nowicka, Marcin Dokowicz, Agnieszka Mańka

**Affiliations:** Faculty of Chemistry, Adam Mickiewicz University, Grunwaldzka 6, 60-780 Poznań, Poland

**Keywords:** Self-avoiding walk, Monte Carlo method, Self-similar and self-affine surface, Fractal dimension

## Abstract

A polymer molecule (represented by a statistical chain) end-grafted to a topologically rough surface was studied by static MC simulations. A modified self-avoiding walk on a cubic lattice was used to model the polymer in an athermal solution. Different statistical models of surface roughness were applied. Conformational entropies of chains attached to uncorrelated Gaussian, Brownian, and fractional Brownian surfaces were calculated. Results were compared with the predictions of a simple analytical model of a macromolecule end-grafted to a fractal surface.

**Electronic supplementary material:**

The online version of this article (doi:10.1007/s00894-012-1546-5) contains supplementary material, which is available to authorized users.

**Figure:**
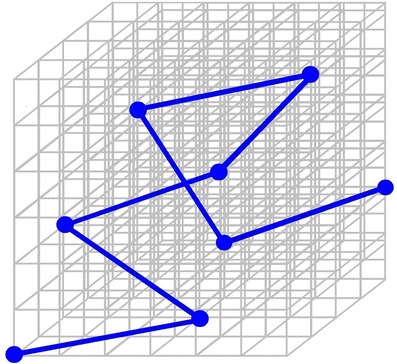
Visualization of SAW generated by the (023) algorithm on a 3D cubic lattice

## Introduction

Surface modification by grafting end-functionalized polymer chains has been extensively used for a variety of applications, including drug delivery [1], molecular electronics [2], cosmetics [3], manufacturing surface-responsive materials [4, 5], biocompatible artificial implants [6], and in many other areas in which colloidal dispersions must be stabilized/destabilized. The properties of polymer layers, which determine the suitability of coatings for particular applications, are strongly dependent on the the grafted chain conformation. In good solvent conditions, the chain conformation is a function of the length of the chain and the density of polymer at the surface [7]. For large polymer densities at the surface, the chains form a structure called the polymer “brush,” whereas they adopt a “mushroom” conformation at low densities. Studies of the conformations of chains that are end-grafted to convex and concave interfaces [8, 9] are relevant to issues such as the deformation of a bacteria cell membrane by an attached chain [10], the application of a macromolecule as a localized pressure microtool [11], or the micromanipulation of individual polymer molecules using AFM [12].

The conformations of polymer chains near surfaces under athermal conditions are governed solely by the excluded volume effects of the polymer itself and the interface. These effects provide the entropic contribution to the total free energy of the grafted chains.

In most studies in which the effect of the excluded volume on the conformations of end-grafted chains was examined, the surface was considered to be homogeneous. However, real solid surfaces contain geometrical irregularities and morphological heterogeneities. These nonuniformities can also influence the conformational behavior of macromolecules that are in close proximity to a surface [13, 14].

In the work described in this paper, we examined the conformation of an isolated polymer molecule (represented by a statistical chain) that is irreversibly attached at one of its ends to a geometrically rough surface. The main aim of the work was to generalize the thermodynamic description of a linear chain that is terminally attached to a homogeneous, flat and purely repulsive surface to that of a chain attached to a rough surface. Note that the present work focuses on the athermal situation. Under purely athermal conditions, the influence of the surface roughness on the chain conformation can be considered to be entirely entropic in nature. The rough surface was modeled based on different types of random geometrical irregularities, including the fractal structure.

## The model

### Generation of a statistical chain

A series of statistically independent samples of linear SAW (self-avoiding walk) chain conformations were generated by means of the static Rosenbluth–Rosenbluth MC approach [15, 16]. Simulations were performed on a 3D regular cubic lattice of lattice constant *a*. In most simulations, the box contained the interface represented by the surface generated by algorithms described in “Generation of the interface.” The method used to generate the SAWs was based on an algorithm in which the segments of the chain were connected by vectors of the type given by the permutation of [0, ±2*a*, ±3*a*] (subsequently referred to as (023) motion). This method is similar to those that have been applied in simulations of polypeptides, where (123) and (023) motions were used [17, 18], and it gives a coordination number *ω* of 24. A high value of *ω* results in very high chain flexibility. The segment length *b* was equal to 3.606*a*. The volume of the simulation box was limited to (601*a*)^3^. Simulations were performed for free (unperturbed) chains and for chains attached to the surface by one end-segment. The surface was rigid and impenetrable to the chain. It was also purely repulsive and there was no chain adsorption except for the irreversible attachment of the terminal segment. Thus, the influence of the surface on the chain conformation was solely entropic in nature.

The model describes the athermal situation, as no interactions except those for the excluded volume of the chain segments and the interface were included (i.e., no long-range and local potentials caused by intermolecular interactions were taken into account) [19].

Chains of up to 100 segments were considered. Each data set presented in the paper was calculated as the average of results obtained from 10^5^ chain conformations.

### Calculation of the chain conformational entropy

The conformational entropy of the SAW chain was calculated by means of the statistical counting (SC) method [20], which is based on the calculation of the quantity $\varpi {\prime_{\text{eff}}},$ defined as

1 $$ \varpi {\prime_{\text{eff}}}(i) = \frac{{\Omega \left( {i + 1} \right)}}{{\Omega (i)}}, $$

where Ω(*i*) is the number of conformations of the chain of *i* segments. The physical meaning of $\varpi {\prime_{\text{eff}}}$ can be related to the effective coordination number of the lattice. For the (023) algorithm with the cubic lattice, this takes a value of 24 for the first segment, and then $\varpi {\prime_{\text{eff}}} \leqslant 23$.

In our study, instead of $\varpi {\prime_{\text{eff}}}$, the average values obtained by the MC sampling method, ${\varpi_{\text{eff}}}$, were used. In practice, ${\varpi_{\text{eff}}}$ was obtained as the number of all empty sites (sites not filled with other segments or the surface) available to a successive segment at each generation step.

The entropy *S* of a chain built of *N* segments was calculated from the equation

2 $$ \frac{S}{{{k_B}}} = \sum\limits_{{i = 1}}^{{N - 1}} {\ln \left( {{\varpi_{\text{eff}}}(i)} \right)}, $$

where *k*_B_ is the Boltzmann constant.

**Fig. 1:**
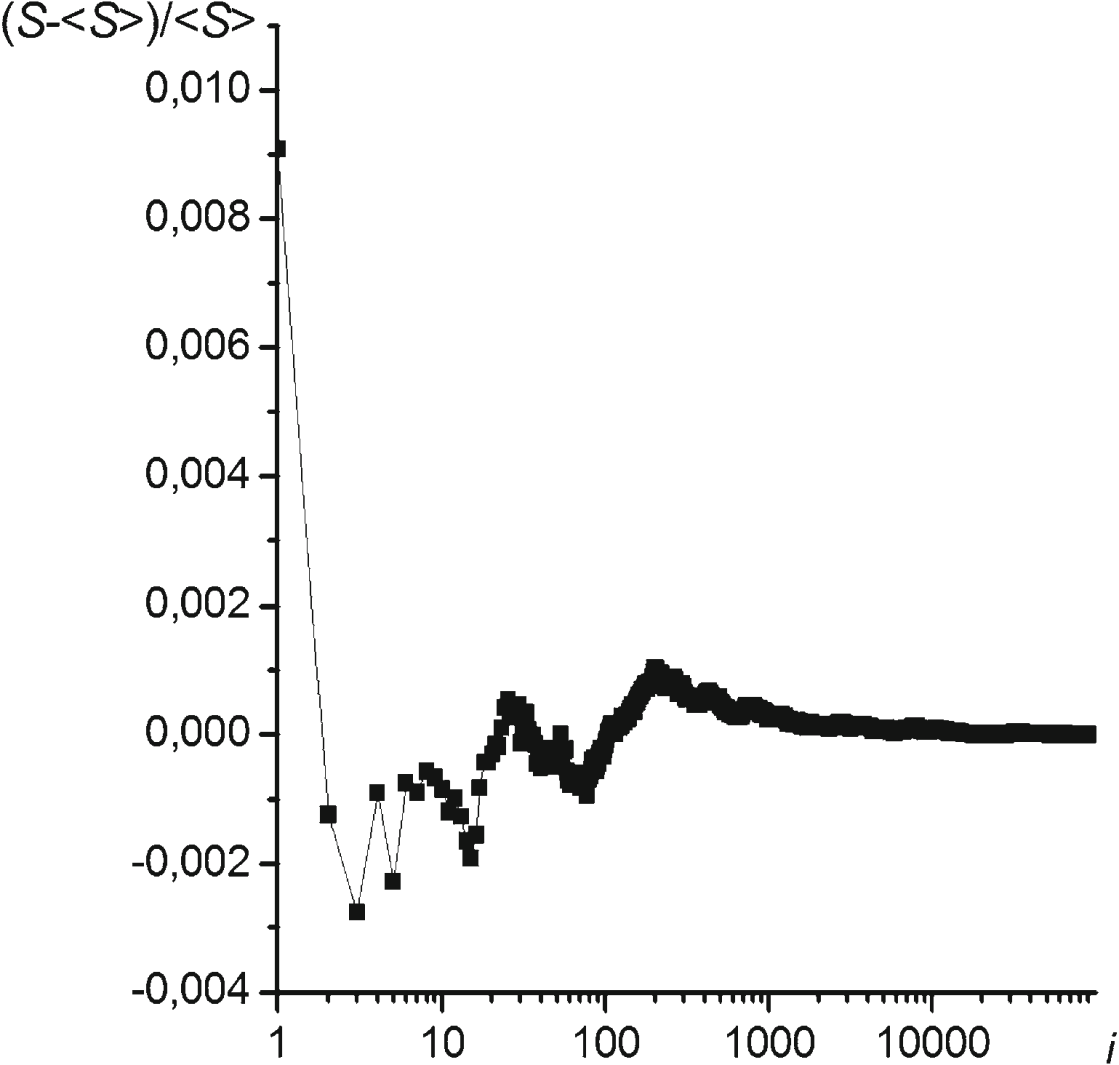
Example of the visualization of the convergence of the conformational entropy of a chain during a simulation (the relative entropy vs. the number of iterations)

The entropy results calculated by means of the SC method rapidly converged to the average (see Fig. 1); the relative standard deviation of the entropy of a chain of 100 segments obtained by 50 independent data sets of 10^5^ conformations was 5.0 × 10^−6^.

### Generation of the interface

The surfaces were generated using two different methods: random displacement (RD) and random midpoint displacement with successive random addition (RMD) [21]. Initially, the generated surface was a square of area (1024*a*)^2^. Before the simulations, the size of the surface was reduced to the length of the edge of the simulation box (601*a*). Three different methods of surface reduction were applied. The methods differed in the way that the altitude of the central point on the surface was chosen: from among all of the surface sites with coordinates between 301 and 723 (sites localized in the central part of the surface), the site that had either (i) the minimum altitude (*z*_min_), (ii) the maximum altitude (*z*_max_), (iii) the elevation closest to the mean altitude of all the surface sites (*z*_mean_), or (iv) a particular random altitude (*z*_random_) was chosen. Chain generation started at a lattice site adjacent to this point.

Since the chain was generated starting at the central point of the simulation box, the method applied produced chains anchored at a local minimum (the valley) or maximum (the apex) of the surface, or at a surface site with the mean or a particular random altitude. The range of possible localizations of the surface site for the chain end attachment was larger than the dimension of the coil formed by the chain (larger than the end-to-end distance and the average radius of gyration for the chain built from *N* = 100 segments). This guaranteed that the surface fragment considered was a statistically significant surface sample containing topological elements (hills and valleys) that were comparable in size to the coil.

In the study, three different types of amorphous surface were examined. The methods used to generate these surfaces are described below.

#### Uncorrelated Gaussian surface

Each uncorrelated Gaussian motion (uGm) surface [21] consisted of elements of the same size as the lattice mesh, and the altitude was determined by the normal distribution. The altitude of each element of the lattice was fully independent of that of its neighboring elements. The uGm surfaces were generated as follows. The elevation *z*_S_ of each lattice site representing the modeled interface was considered to be the integer part of the variable of the normal distribution defined by the expression

3 $$ {z_{\text{S}}} = {z_0} + \sigma \sqrt {{ - 2\ln (X)}} \cos \left( {2\pi \,X} \right), $$

where *z*_0_ is the assumed mean altitude of the surface, *σ* is the standard deviation of the altitude, and *X* is a variable representing a uniform distribution in the range [0,1). All sites with altitude coordinates *z* < *z*_S_, which are located inside the solid phase, were marked as inaccessible to the chain.

#### Brownian motion and fractional Brownian motion surfaces

Brownian motion (Bm) surfaces and fractional Brownian motion (fBm) surfaces were generated by means of the RMD method. Surface generation consisted of modifying the initially smooth surface described by *z* = *z*_0_, the initial mesh size of which was equal to 1024*a*. The modification led to a recursive reduction in the mesh size by a factor of 2 and a change in the altitude of a randomly chosen square by an increment whose standard deviation was given by a factor of 2^*H*^, where *H* is the Hurst exponent [21]. For the floating point number representation of the surface altitude, the exponent *H* is directly related to the fractal dimension *D*_F_ of the surface as follows:

4 $$ H = 3 - {D_{\text{F}}}. $$

At random intervals, all of the altitudes were subjected to random displacements in order to minimize artefacts of the surface construction. The method applied guarantees scale invariance in the range between the lattice constant and the length of the simulation box, since the fractal irregularities of the surface do not have amplitude and their dimensions increase with the object scale. This method produced Bm surfaces at *H* = 1/2 and fBm surfaces at *H* ≠ 1/2. At the end of surface generation, the resulting altitudes *z* were converted to discrete values. All sites with coordinates *z* < *z*_S_ were marked as inaccessible to the chain.

### Characterization of surfaces

The last step in both of the methods of surface generation described above was to convert the results given as floating point numbers to integers. Thus, the final values of the parameters describing the surface roughness differed from the generated ones. In order to characterize the roughness of surfaces described by integer altitudes, the standard deviation of these altitudes and the “empirical” fractal dimension were applied. The empirical fractal dimension was calculated as follows:

5 $$ {D_{\text{F}}} = \frac{{\ln (X)}}{{\ln (L)}}, $$

where *X* is the total surface area, which includes the upper and side wall areas of the surface elements, and *L* is the edge length of the square obtained by projecting the surface sample onto the wall of the simulation box. The parameter *D*_F_ takes a value of 2 for a smooth flat surface and tends to 3 for an extremely rough surface.

## Results

### Analysis of the surface roughness

**Fig. 2:**
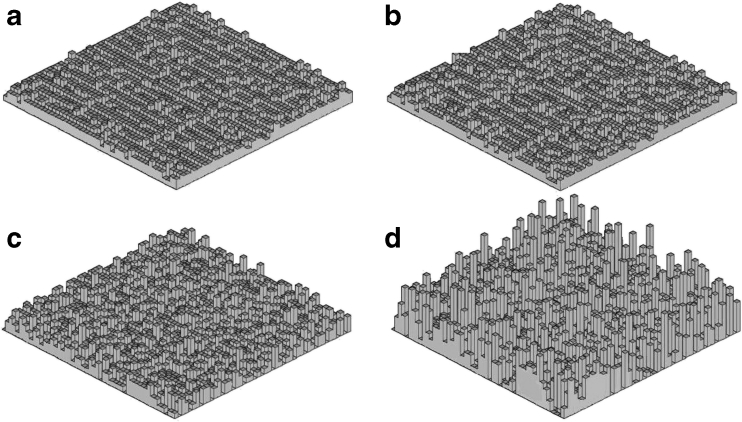
Samples of uGm surfaces with different standard deviations of altitude. Surface roughness parameters for** a**–**d** are collected in Table 1

The images of a few examples of uGm surfaces generated for different standard deviations *σ* of the altitude are shown in Fig. 2. As seen, increasing *σ* changes the surface shape from planar (Fig. 2a) to a brush with a large number of sharp apices and deep and narrow valleys (Fig. 2d and Table 1).

**Table 1:** Roughness parameters for the surfaces depicted in Figs. 2 and 4

Figure	Surface type	*H*	*σ*	*D*_F_
2a	uGm	–	0.4	2.089
2b	uGm	–	0.5	2.114
2c	uGm	–	1.0	2.184
2d	uGm	–	3.0	2.320
4a	Bm	0.5	1.0	2.109
4b	Bm	0.5	2.0	2.173
4c	Bm	0.5	3.0	2.215
4d	fBm	0.01	1.0	2.240
4e	fBm	1.0	1.0	2.055
4f	fBm	2.0	1.0	2.014

**Fig. 3:**
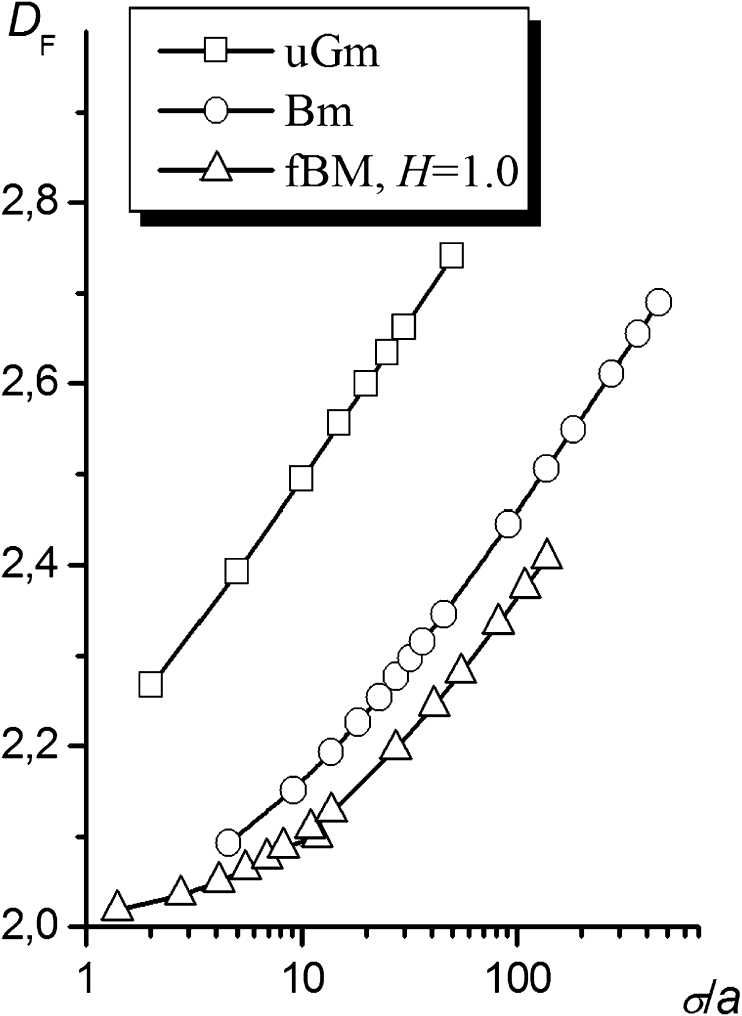
The dependencies of the fractal dimensions of uGm, Bm, and fBm surfaces on the standard deviation of altitude

Although the uGm surfaces are not fractal objects, we have adopted their fractal dimension as a measure of their roughness. The fractal dimensions of the surfaces, *D*_F_, versus the standard deviations of altitude, *σ*, calculated for all of the surface types used in the study are shown in Fig. 3. As follows from this figure, increasing *σ*/*a* leads to an increase in *D*_F_ from 2 (the flat surface) toward 3. The *D*_F_ = log(*σ*/*a*) dependencies have the same slope irrespective of the surface type for high values of *σ*/*a*.

**Fig. 4:**
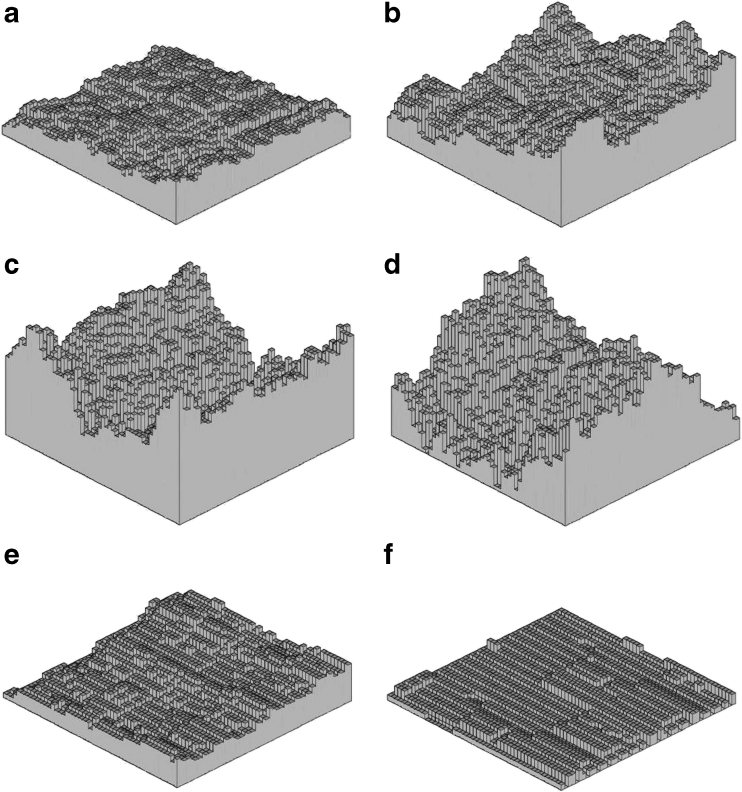
Samples of Bm (**a**–**c**) and fBm (**d**–**f**) surfaces generated by the RMD method. Surface roughness parameters for** a**–**f** are collected in Table 1

Figure 4 presents examples of Bm and fBm surfaces. As seen, the fractal Bm surfaces (Fig. 4a–c) are good models of amorphous surfaces with different roughnesses. Increasing the Hurst coefficient of the fBm surface (Fig. 4d–f) implies a transition from an amorphous surface to a surface morphology close to that of the crystal fracture surface.

The applied methods of simulation permit the generation of interfaces with different topologies: from amorphous surfaces to regular ones corresponding to crystal fracture, and from smooth surfaces to rough brush structures like those obtained by modifying the interface through polymer adsorption.

### Conformational entropy of a grafted chain

The conformational entropy of a free unperturbed linear chain can be calculated from Eq. 6, which was derived on the basis of renormalization group theory [22, 23]:

6 $$ \frac{S}{{{k_{\text{B}}}}} = \ln \left( {{C^{\text{F}}}} \right) + \left( {{\gamma^{\text{F}}} - 1} \right)\ln \,N + N\,\ln \left( {\omega_{\text{eff}}^{\text{F}}} \right), $$

where *C* and the average effective coordination number of the lattice *ω*_eff_ are constants that depend on the geometrical details of the model (namely on the coordination number of the lattice), and *γ* is the universal constant, which takes the value 7/6 for a free unperturbed chain in a 3D lattice. The superscript F is used to denote the free unperturbed chain. The *S* = f(*N*) relationship represented by Eq. 6 was compared with that obtained for the model in which long-range excluded volume effects are not incorporated [the nonreversal random walk (NRRW)], which is given by [24]

7 $$ \frac{S}{{{k_{\text{B}}}}} = \ln \left( \omega \right) + N\,\ln \left( {\omega - 1} \right), $$

where *ω* is the coordination number of the lattice. When one compares Eqs. 6 and 7 it becomes apparent that the parameter *C* ^F^ in Eq. 6 refers to the initial part of macromolecule (i.e., the part generated at the very beginning). It can be correlated to the number of possible positions of the second segment around the first one, and can be related to the term ln(*ω*) in the NRRW model. Moreover, one can conclude from the above comparison that the parameter *γ* ^F^ as well as the difference between $\omega_{\text{eff}}^{\text{F}}$ and *ω*−1 describe the excluded volume effects in the more distant parts of the chain.

Anchoring the chain end to a surface implies a reduction in the conformational entropy of the macromolecule. Later in the text, this reduction in the chain entropy following its attachment to a surface is referred to as the “entropy of chain anchoring” Δ*S*. If the anchoring takes place on a planar smooth surface, Δ*S* is related to the segment number by the following relationship (based on Eq. 6):

8 $$ - \frac{{\Delta S}}{{{k_{\text{B}}}}} = - \frac{{{S^{\text{A}}} - {S^{\text{F}}}}}{{{k_{\text{B}}}}} = \ln \left( {\frac{{{C^{\text{A}}}}}{{{C^{\text{F}}}}}} \right) + \left( {{\gamma^{\text{A}}} - {\gamma^{\text{F}}}} \right)\ln \,N + N\,\ln \left( {\frac{{\omega_{\text{eff}}^{\text{A}}}}{{\omega_{\text{eff}}^{\text{F}}}}} \right), $$

where the index A refers to the anchored chain. For a long chain, the ratio *C* ^A^/*C* ^F^ is much lower than *N* and we can neglect the first term on the right hand side of Eq. 8. Moreover, the probability that the segments will make contact with the surface diminishes as their distance from the grafted segment (measured along the chain) increases, since the anchored chain most likely propagates towards the bulk, where the conditions are similar to those of the free chain. Hence, it can be assumed that the ratio ${{{\omega_{\text{eff}}^{\text{A}}}} \left/ {{\omega_{\text{eff}}^{\text{F}} \approx 1}} \right.}$ and Eq. 8 takes the form:

9 $$ \frac{{\Delta S}}{{{k_{\text{B}}}}} = \left( {{\gamma^{\text{A}}} - {\gamma^{\text{F}}}} \right)\ln \,N = \Delta \gamma \ln N , $$

where Δ*γ* ≈ −0.47, since the exponent *γ* ^A^ is equal to 0.70 ± 0.02 for the planar surface [23, 25]. In the case of chains that are end-attached to a smooth planar surface, Eq. 9 was found to provide a good approximation of the Δ*S* = f(*N*) dependence [23, 25].

**Fig. 5:**
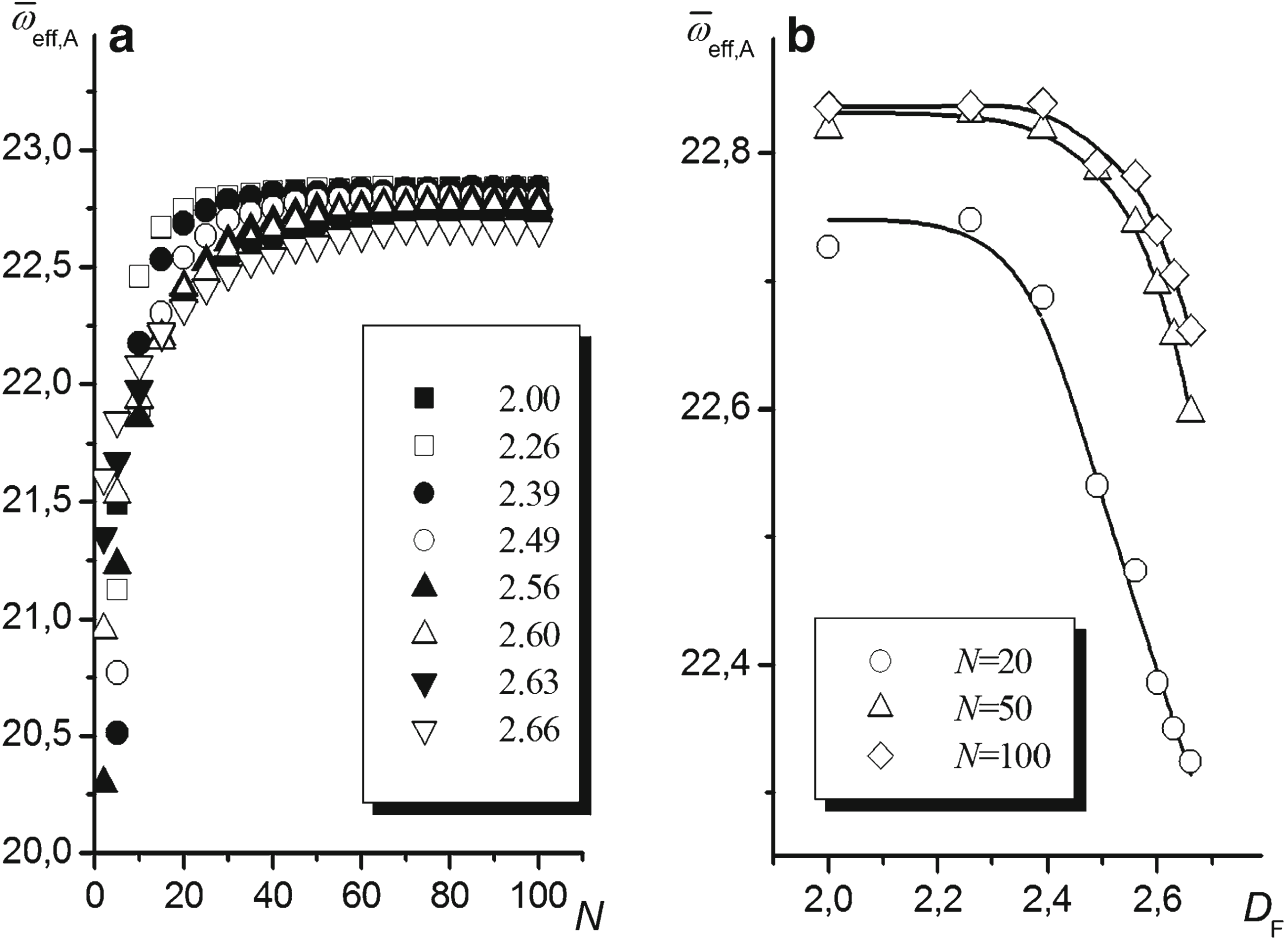
Effective lattice coordination number versus **a** the length of the chain for chains anchored to uGm surfaces with different degrees of roughness (values of *D*_F_ are indicated in the figure) and **b** the fractal dimension of the surface for selected chain lengths (values of *N* are marked in the figure). The chain is attached at *z* = *z*_random_. The plot of ${\varpi_{\text{eff}}}$ vs.* σ* is shown in the “Electronic supplementary material” (ESM)

In order to assess the applicability of Eq. 9 to chains anchored to rough surfaces, the obtained dependencies of the effective coordination number (used in this work to calculate the absolute entropies *S* ^F^ and *S* ^A^) on the chain length and the fractal dimension of the surface were analyzed. Examples of the dependencies of ${\varpi_{\text{eff}}}$ on *N* for chains anchored at *z* = *z*_random_ on uGm surfaces characterized by different values of *D*_F_ are shown in Fig. 5a. The plot of ${\varpi_{\text{eff}}} = {\text{f}}(N)$ starts from ${\varpi_{\text{eff}}}$ close to 16, corresponding to the number of free sites near a segment located at an altitude *z*_S_ + 1 (${\varpi_{\text{eff}}} = 6$ is reached only for a planar surface), then it increases asymptotically, reaching a value close to 22.9 at *N* = 100. The maximum coordination number of the lattice *ω* = 23 (resulting from the NRRW assumption) is not reached because of the excluded volume effect. Figure 5b presents the dependence ${\varpi_{\text{eff}}} = {\text{f}}\left( {{D_{\text{F}}}} \right)$. As expected, an increase in the surface roughness causes a decrease in the effective coordination number, especially for short chains.

**Fig. 6:**
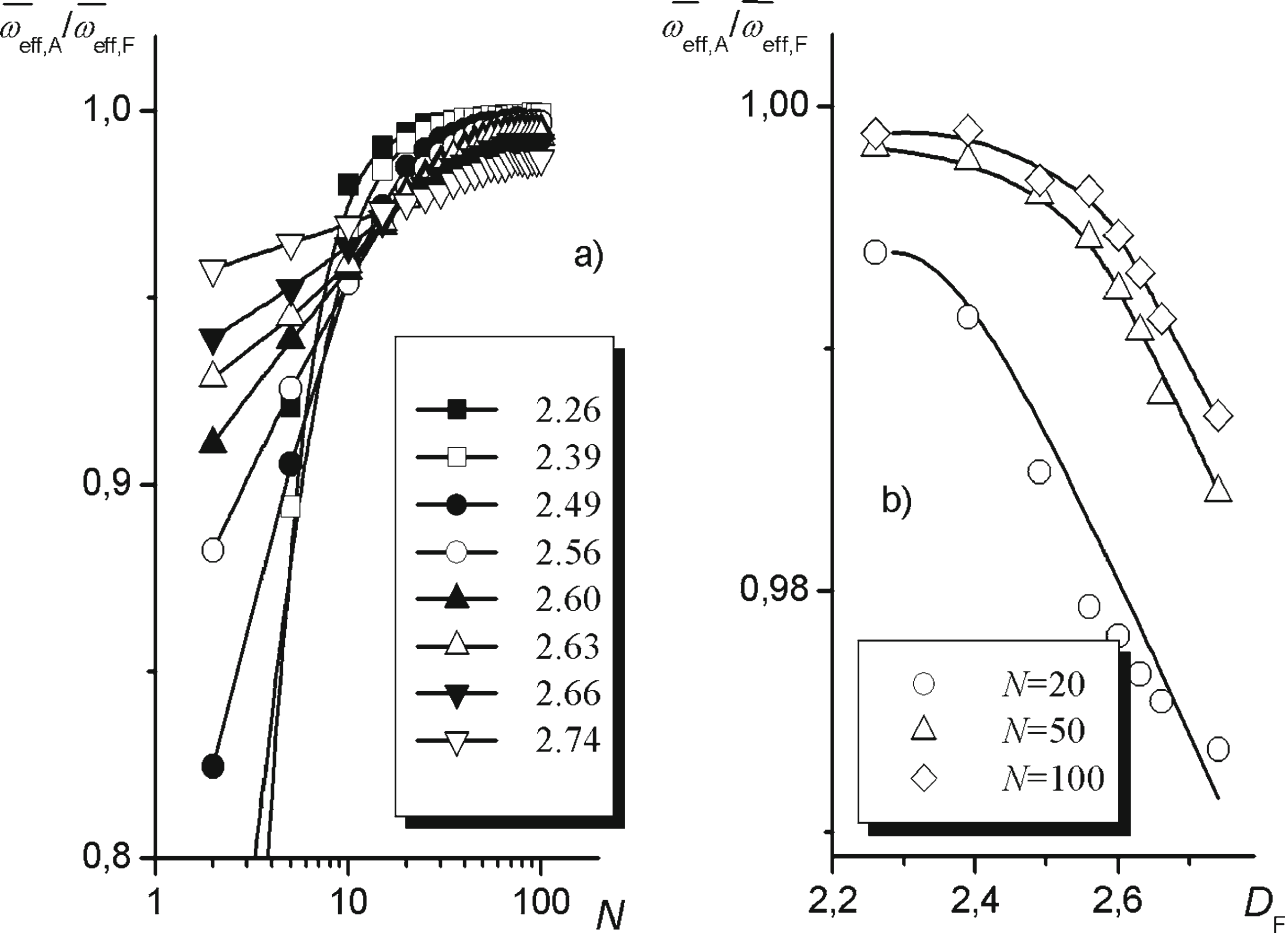
The dependencies **a** ${{{{\varpi_{{{\text{eff,}}\,{\text{A}}}}}}} \left/ {{{\varpi_{{{\text{eff,}}\,{\text{F}}}}}}} \right.}$ vs.* N* and **b** ${{{{\varpi_{{{\text{eff,}}\,{\text{A}}}}}}} \left/ {{{\varpi_{{{\text{eff,}}\,{\text{F}}}}}}} \right.}$ vs.* D*_F_ calculated for uGm surfaces (*D*_F_ and *N* values are indicated in the figure, *z* = *z*_random_). The plot of ${{{{\varpi_{{{\text{eff,}}\,{\text{A}}}}}}} \left/ {{{\varpi_{{{\text{eff,}}\,{\text{F}}}}}}} \right.}$ vs.* σ* is shown in the ESM

Changes in the effective coordination number with *N* can also be illustrated using a different coordinate system. Figure 6a presents the dependence of the ratio ${{{\varpi_{\text{eff}}^{\text{A}}}} \left/ {{\varpi_{\text{eff}}^{\text{F}}}} \right.}$ on the segment number *N*, calculated for uGm surfaces at *z* = *z*_random_. The dependencies obtained for other types of surfaces (i.e., Bm and fBm) and different initial values of the parameters are similar in shape: as *N* increases, the ratio ${{{\varpi_{\text{eff}}^{\text{A}}}} \left/ {{\varpi_{\text{eff}}^{\text{F}}}} \right.}$ tends asymptotically to a certain value that depends only slightly on the fractal dimension of the surface. However, at low *N*, a pronounced effect of the value of *D*_F_ on the ${{{\varpi_{\text{eff}}^{\text{A}}}} \left/ {{\varpi_{\text{eff}}^{\text{F}}}} \right.}$ ratio is observed, indicating that linear dependence (9) is not a good approximation of the real Δ*S* = f(*N*) relationship for rough surfaces—at least not in the examined range of *N* values, where neglecting the term ${{{{C^{\text{A}}}}} \left/ {{{C^{\text{F}}}}} \right.}$in Eq. 8 seems to be an oversimplification. Figure 6b indicates that an increase in the surface roughness implies a decrease in the relative coordination number of the lattice.

**Fig. 7:**
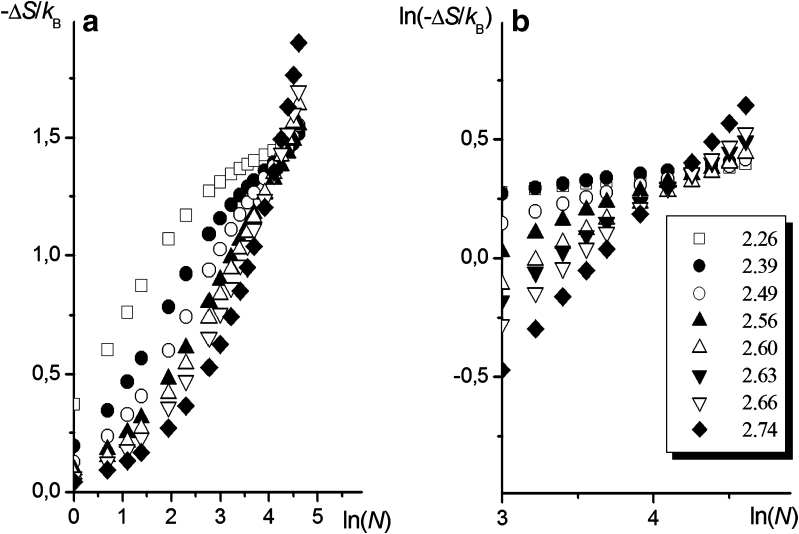
The linear-logarithmic (**a**) and logarithmic-logarithmic (**b**) dependencies of the conformational entropy vs. the segment number calculated for uGm surfaces at different *D*_F_ values (as indicated in the figure); *z* = *z*_random_

Figure 7a presents the Δ*S* = f(*N*) dependencies obtained from our simulations of uGm surfaces at *z* = *z*_random_. As expected, the dependencies are not linear, but they become linear on a log-log scale, especially for relatively long chains, which points to the predominant effect of the third term in Eq. 8 (determined by the ratio of the effective coordination numbers) on the shapes of these dependencies. In the case of extremely irregular surfaces, the ${ \ln }\left( {-\Delta S} \right) = {\text{f}}\left( {{ \ln }(N)} \right)$ dependencies become linear across the whole range of *N* and mutually intersect in a very small region of *N* values (Fig. 7b). Based on the linear character of the relationships studied, the parameters *A* and *ζ* in the following equation:

10 $$ - \frac{{\Delta S}}{{{k_{\text{B}}}}} = A{N^{\zeta }} $$

were chosen to characterize the influence of the surface roughness on the entropy of chain anchoring (Δ*S*).

### The effect of surface topology on the entropy of chain anchoring

The results of simulations concerning the effect of surface roughness on the conformational entropy of end-attached chains are now presented and discussed separately for each of the three types of surface models described above, which mimic the most common real surface geometries.

#### Brush-like interfaces

**Fig. 8:**
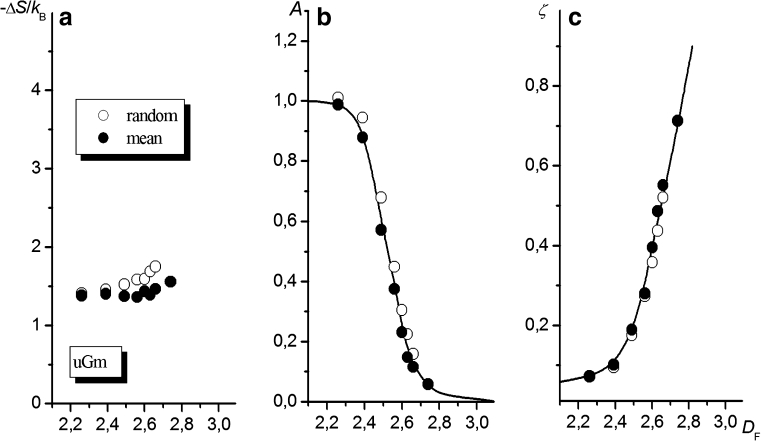
Macromolecule tethered to the uGm surface. The effects of the fractal dimension of the surface, *D*_F_, on Δ*S* (**a**) and the coefficients* A* and *ζ* from Eq. 10 (**b** and **c**, respectively) are shown. Values obtained for different altitudes of the tethered segment are marked with different* symbols*, as indicated in the figure (plots of Δ*S*,* A*, and* ζ* vs. *σ* are shown in the ESM)

The dependencies of Δ*S* and the parameters *A* and *ζ* and on the fractal dimension of the uGm surface for a chain consisting of 100 segments are shown in Fig. 8. As follows from Fig. 8a, the *D*_F_ value affects the chain entropy only slightly. Also, the results show only a minor dependence on whether the attachment point was chosen at random or the chain attachment was forced to occur at the point of mean altitude.

It is clear that the influence of *D*_F_ on Δ*S*, as observed in Fig. 8a, is minimal, considering that the results presented here are for a chain with *N* = 100 (i.e., *N* in the vicinity of the range of *N* in which the dependencies of ln(−Δ*S*) vs. ln(*N*) intersect, as shown in Fig. 7b and mentioned in the previous section). The slopes and initial intercepts of the plots of ln(−Δ*S*) vs. ln(*N*) in Fig. 7b are strongly influenced by *D*_F_. The appearance of a region in which the plots intersect (as well as the existence of a critical value for the fractal dimension of the surface, as discussed further in “Self-affine surfaces”) is probably a result of discrete properties of the network, as governed by the constants *a* and *b* that characterize the grained structure of the system.

As illustrated in Fig. 8b and c, as the fractal dimension of the uGm surface increases, the value of the parameter *A* decreases to zero while the value of another parameter in Eq. 10,* ζ*, increases without restriction (at least in the range of *D*_F_ values examined). No distinct dependence of either *A* or *ζ* on the chosen chain attachment point on the surface was observed.

The decrease in *A* with increasing *D*_F_ probably results from the greater conformational freedom of the initial part of the macromolecule (i.e., the part composed of segments generated at the very beginning, which is in close proximity to the end segment attached to the surface), since the spatial hindrance due to the presence of the surface is expected to decrease as *D*_F_ increases. This can be understood when one considers that the attachment of very short chains to the flat surface involves eliminating approximately half of all possible conformations (since a half-space is blocked by the surface). Anchoring the chain to a surface with a large fractal dimension corresponds to placing its end in the “brush” area, where the number of possible trajectories for the chain increases (since those between the brush rods are possible too). On the other hand, analysis of the influence of *D*_F_ on the value of *ζ* (Fig. 8c) indicates that, for high *D*_F_, the probability of encountering a lattice site belonging to the surface increases with increasing chain length. Therefore, the reduction in the conformational entropy becomes larger and occurs more rapidly as the chain length increases. This can be understood by noting that, as *D*_F_ increases, the depth of a valley in which the chain can occur also increases, forcing the chain to adopt a more extended conformation. In the extreme case (i.e., for very high *D*_F_), the number of possible chain conformations decreases to that corresponding to the chain placed among parallel rods, and the drop in entropy becomes proportional to the chain length, which implies that the exponent *ζ* tends to unity:

11 $$ \mathop{{\lim }}\limits_{{{D_{\text{F}}} \to 3}} \zeta = 1. $$

#### Self-similar surfaces

The Bm surface is locally smoother then the uGm one since there are no narrow slits. Therefore, the frequency of violation of the SAW constraint was smaller (a smaller number of trial conformations was rejected) with the Bm surface, so simulation results were obtained much more easily than for the chain bound to the uGm surface.

**Fig. 9:**
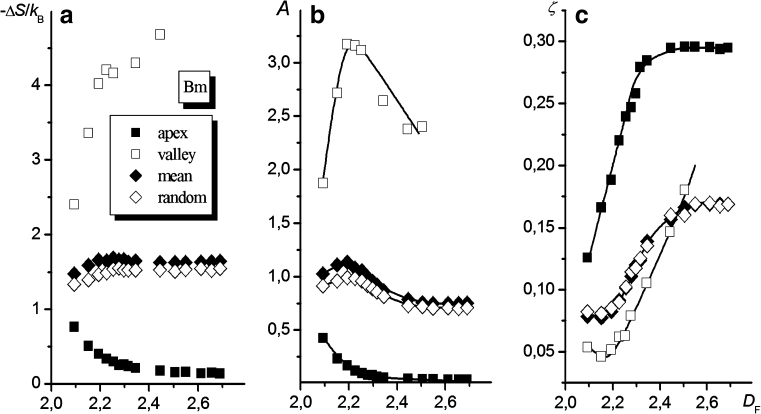
Macromolecule tethered to the Bm surface. The dependencies for the same three pairs of variables (**a**–**c**) as shown in Fig. 8, determined for different altitudes of the tethered segment (plots of Δ*S*,* A*, and* ζ* vs. *σ* are provided in the ESM) are shown

Figure 9 presents the results obtained for four different locations of the chain end (among which the first three were imposed): at the apex, at the valley bottom, at the surface element of mean altitude, and at the altitude *z* = *z*_random_. As seen (Fig. 9a), anchoring the chain to the apex of a local irregularity results in only a small reduction in its conformational entropy, which decreases with increasing fractal dimension of the surface, while anchoring the chain to the bottom of a local depression brings about a significant drop in the entropy, which increases with the depth of the depression (valley). These results reflect the fact that both the apex height and the valley depth increase with rising *D*_F_. In the case of forced chain attachment at *z* = *z*_mean_ (that is, at the slope of a local elevation), the entropies of chain anchoring change only slightly as the fractal dimension of the surface is varied, and their values are somewhat higher than the corresponding ones found for a chain attached at the point with altitude *z* = *z*_random_. This can be understood by realizing that in the latter case the chain “prefers” to attach to the surface at a point with a high *z* value, due to the smaller number of rejected trial conformations in the simulation process.

The *A* and* ζ* vs. *D*_F_ relationships are even more complex than Δ*S =* f(*D*_F_). As can be seen from Fig. 9b and c, in most cases (i.e., for *z* = *z*_valley_, *z*_mean_, *z*_random_), the two dependencies show opposite tendencies: parameter *A* passes through its maximum value and parameter *ζ* attains its minimum value in practically the same range of *D*_F_ values. However, when the chain is anchored in the valley, the unlimited increase in the exponent *ζ* is accompanied by a decrease in the coefficient *A* to zero as *D*_F_ increases, just as seen for the uGm surface. The exponents calculated for chains anchored at all of the other anchoring sites considered tend to a constant value ζ*. This value depends on the anchoring site of the chain; when the chain end is anchored at the site with altitude *z* = *z*_apex_, this value is equal to 0.29 ± 0.02, whereas for the two other anchoring locations (*z*_random_ and *z*_mean_), ζ* = 0.17 ± 0.01. In all three cases, the lowest value of *D*_F_ at which *ζ* = ζ*, subsequently denoted $D_{\text{F}}^{\text{crit}}$, is equal to about 2.5.

#### Self-affine surfaces

**Fig. 10:**
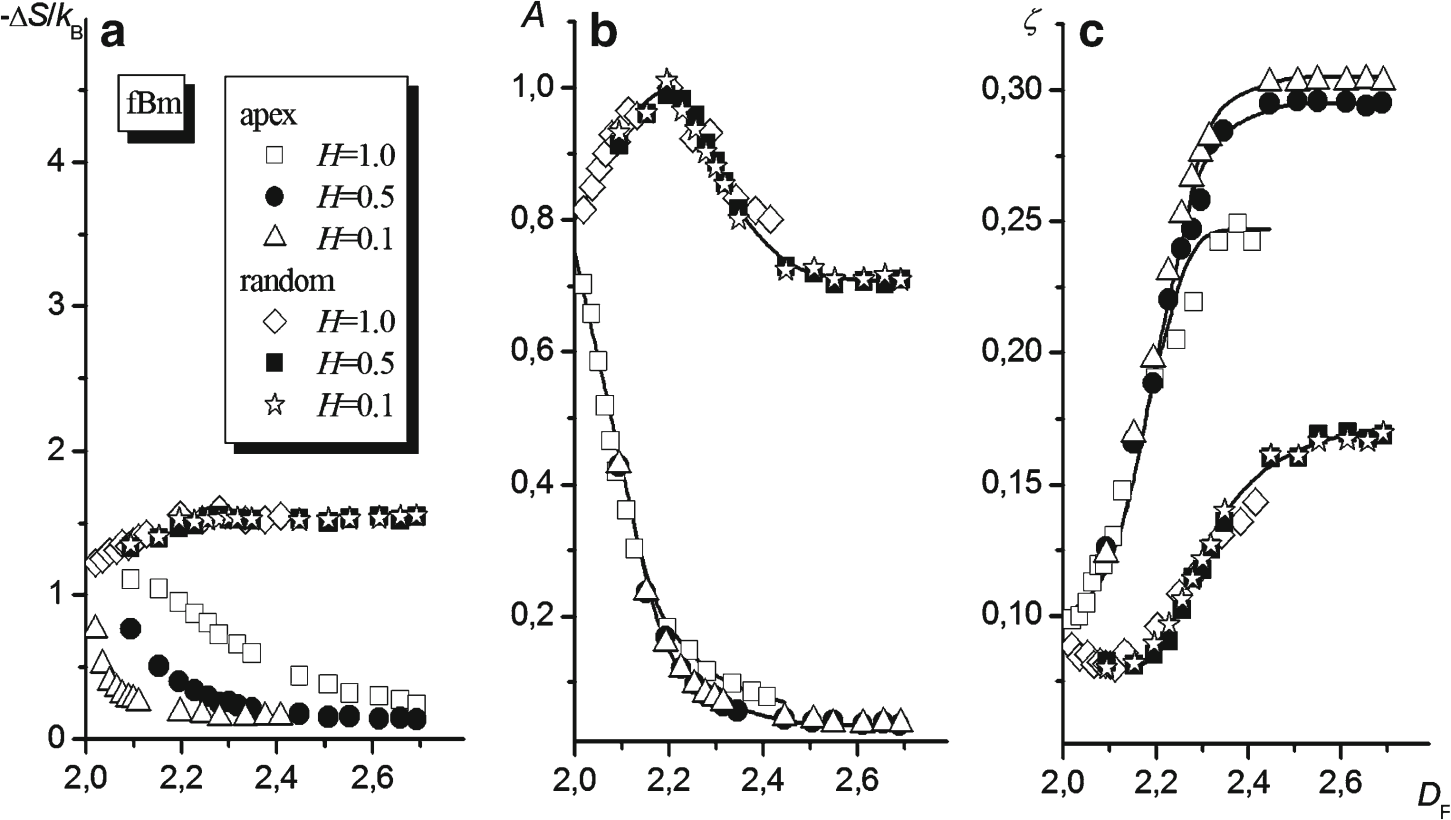
Macromolecule tethered to the fBm surface. The dependencies of the entropy of chain anchoring (**a**), the coefficient *A* (**b**), and the exponent *ζ* (**c**) on the fractal dimension of the surface *D*_F_ are shown. Results obtained for different altitudes and different Hurst coefficients are marked with different* symbols*, as indicated in the figure (plots of Δ*S*,* A*, and* ζ* vs. *σ* are shown in the ESM)

fBm surfaces have more general properties than Bm ones. Namely, their roughness is determined by the Hurst parameter, *H*, besides the standard deviation of the altitude. The higher the value of *H*, the smoother the surface. Figure 10a shows the entropies of anchoring for chains with *N* = 100 anchored at two different points (i.e., *z*_random_ and *z*_apex_) on the fBm surface, calculated for various *D*_F_ and *H* values. As seen, the dependencies of Δ*S* on *D*_F_ are monotonic for both locations of the anchored segment and all examined values of *H*. However, for a chain anchored to a randomly chosen element on the fBm surface, the values of Δ*S* are practically independent of the Hurst parameter and grow slowly with the fractal dimension of the surface, whereas for a chain anchored to the apex of local elevation, the *H* value affects the entropy change and smaller Hurst parameter values (i.e., rougher surfaces) cause steeper falls in Δ*S* with increasing *D*_F_. Figures 10b and c illustrate the influence of *D*_F_ on the coefficient *A* and the exponent *ζ* for chains anchored at *z*_random_ and *z*_apex_. In the former case (*z* = *z*_random_), just as seen for the Bm surface, the dependence of *A* on *D*_F_ passes through a maximum regardless of the value of the Hurst parameter, whereas the exponent *ζ* tends to the value 0.17 ± 0.01 with increasing *D*_F_. In the latter case (*z* = *z*_apex_), increasing *D*_F_ causes *A* to decrease and, as in the previous case, *ζ* to increase to a certain constant value, which is dependent (albeit weakly) on the Hurst parameter; ζ* shifts from 0.30 to 0.27 when *H* changes from 0.1 to 1.0. For both of the examined locations of the attachment point at the fBm surface $D_{\text{F}}^{\text{crit}} \cong {2}.{5}$, which is similar to that found earlier for the Bm surface.

The shape of the dependence of *ζ* on *D*_F_ points to the existence of two different scale regimes of compatibility between two fractal objects—the fractal surface and the fractal coil. The fractal dimension $D_{\text{F}}^{\text{crit}}$ of the transition between two regimes corresponds to the standard deviation of the surface altitude, equal to ${{\sigma } \left/ {a} \right.} \cong {1}00\pm {2}0$ (see Fig. 3). On the other hand, the characteristic size of the coil—i.e., the root-mean-square end-to-end distance, *R*_H_, defined by the following equation:

12 $$ \frac{{{R_{\text{H}}}}}{a} = \frac{b}{a}{N^v}, $$

where *v* denotes the Flory exponent, which is equal to 3/5 for the athermal solution—equals about 106 if the coil’s anisotropy is taken into account (i.e., the ratio of the distances between chain ends, measured along the coordinate axes, was taken to be $2\sqrt {6} :2:1$), and a perpendicular orientation of the longest axis with respect to the surface is assumed. At ${D_{\text{F}}} = D_{\text{F}}^{\text{crit}}$, the characteristic sizes of both fractal objects (i.e., the macromolecule and the fragment of the surface occupied by it) become comparable. For ${D_{\text{F}}} > D_{\text{F}}^{\text{crit}}$, the entropy of chain anchoring Δ*S* scales with the chain length for a constant value of the exponent *ζ* = ζ*. For ${D_{\text{F}}} < D_{\text{F}}^{\text{crit}},$ the relationship Δ*S* vs. *N* becomes dependent on the specific properties of the lattice model used for the simulation: if the linear dimensions of the considered quasi-fractal objects tend to the value of the lattice constant, the self-similarity of these objects disappears. This conclusion may have more general relevance—one can extend it to real objects whose characteristic linear dimensions decrease to those of atomic ones.

Another indication of this issue, as already mentioned in “Brush-like interfaces,” seems to be the occurrence of a region where all of the Δ*S* vs. *N* curves obtained for different fractal dimensions of the uGm surface intersect, which can be explained by the fact that the characteristic size of surface irregularities and the characteristic size of the macromolecule are governed by the grain size of the surface roughness.

### Simple analytical model of a chain terminally anchored to the fractal surface

In order to interpret the results of the MC simulations, we performed an additional analysis of the influence of surface roughness on the conformational entropy of an end-grafted chain. In this analysis, we took into account the fact that when a chain is anchored to an impenetrable surface a number of possible chain conformations are eliminated. This elimination can be considered to be part of the segment density distribution in a free coil being cut off by this very surface.

The radial segment density distribution around the center of mass of the coil formed by the unperturbed SAW chain can be approximately expressed by [26]

13 $$ {\rho_{\text{F}}}(r) = {\rho_{\text{G}}}(r){\rho_{\text{E}}}(r) = BN{\left( {\frac{r}{b}} \right)^2}\exp \left( { - \frac{9}{N}{{\left( {\frac{r}{b}} \right)}^2}} \right)\exp \left( { - \frac{1}{{72}} \cdot \frac{{{N^2}{a^3}}}{{{r^3}}}} \right), $$

where *r* = (*x* ^2^ + *y* ^2^ + *z* ^2^)^1/2^, *x*, *y*, and *z* are the Cartesian coordinates and *B* is the normalization constant. The term *ρ*_G_(*r*) refers to the Gaussian distribution, whereas *ρ*_E_(*r*) corresponds to the excluded volume effect related to the segment volume *a* ^3^.

Let’s assume that when the end-segment of the macromolecule is attached to the surface at the site of mean altitude (i.e.,* z* = *z*_mean_), the center of the coil formed by the remaining segments is located at a distance from the attachment site equal to its average gyration radius, *R*_G_. Since the surface is rough, the cut-off of distribution (13) should be expressed by the cumulative distribution function *ρ*_S_(*z*) associated with the assumed altitude distribution of the surface elements. Assuming that the distribution is Gaussian, the cumulative distribution of lattice sites not occupied by the surface elements reads:

14 $$ {\rho_S}(r) = \frac{1}{2}\left( {1 + {\text{erf}}\left( {\frac{{ - \left( {x - {R_{\text{G}}}} \right)}}{{\sqrt {{2 }} \sigma }}} \right)} \right). $$

Finally, the distribution of the segments in the coil formed by the terminally attached chain takes the form

15 $$ {\rho_{\text{A}}}(r) = {\rho_{\text{F}}}(r){\rho_{\text{S}}}(z). $$

**Fig. 11:**
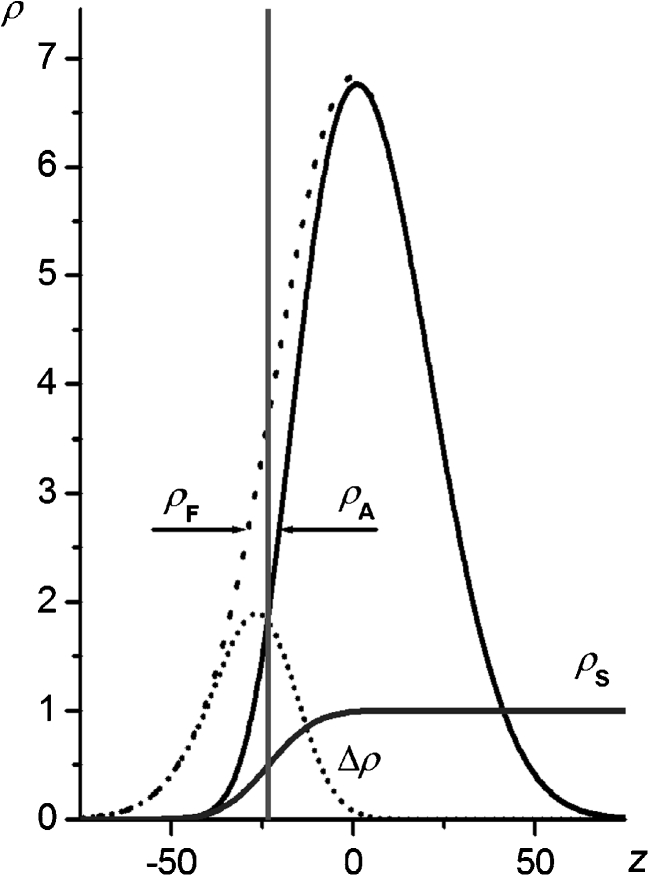
Probability distributions for finding a segment in the unperturbed coil (*ρ*_F_) and in the terminally attached chain (*ρ*_A_), and the cumulative distribution of free sites near the surface (*ρ*_S_). The difference Δ*ρ* = *ρ*_F_ −* ρ*_A_ is also indicated. The *vertical line* denotes the mean elevation of the surface (*z*_mean_); *N* = 100; *σ*/*a* = 10

The segment density distributions in the free unperturbed coil and the coil terminally attached to the fractal surface, as calculated from Eqs. 13 and 15, respectively, are shown in Fig. 11. As shown, attachment deforms the initial distribution (13). The deformation is equivalent to the elimination of some conformations of the chain. Assuming that the conformational entropy of the chain can be related to the product *ρV*, where *V* is the volume occupied by the macromolecule, the reduction in conformational entropy due to chain attachment can be calculated from

16 $$ \frac{{\Delta S}}{{{k_{\text{B}}}}} = \ln \left( {\frac{{\int {{\rho_{\text{A}}}dr} }}{{\int {{\rho_{\text{F}}}dr} }}} \right). $$

**Fig. 12:**
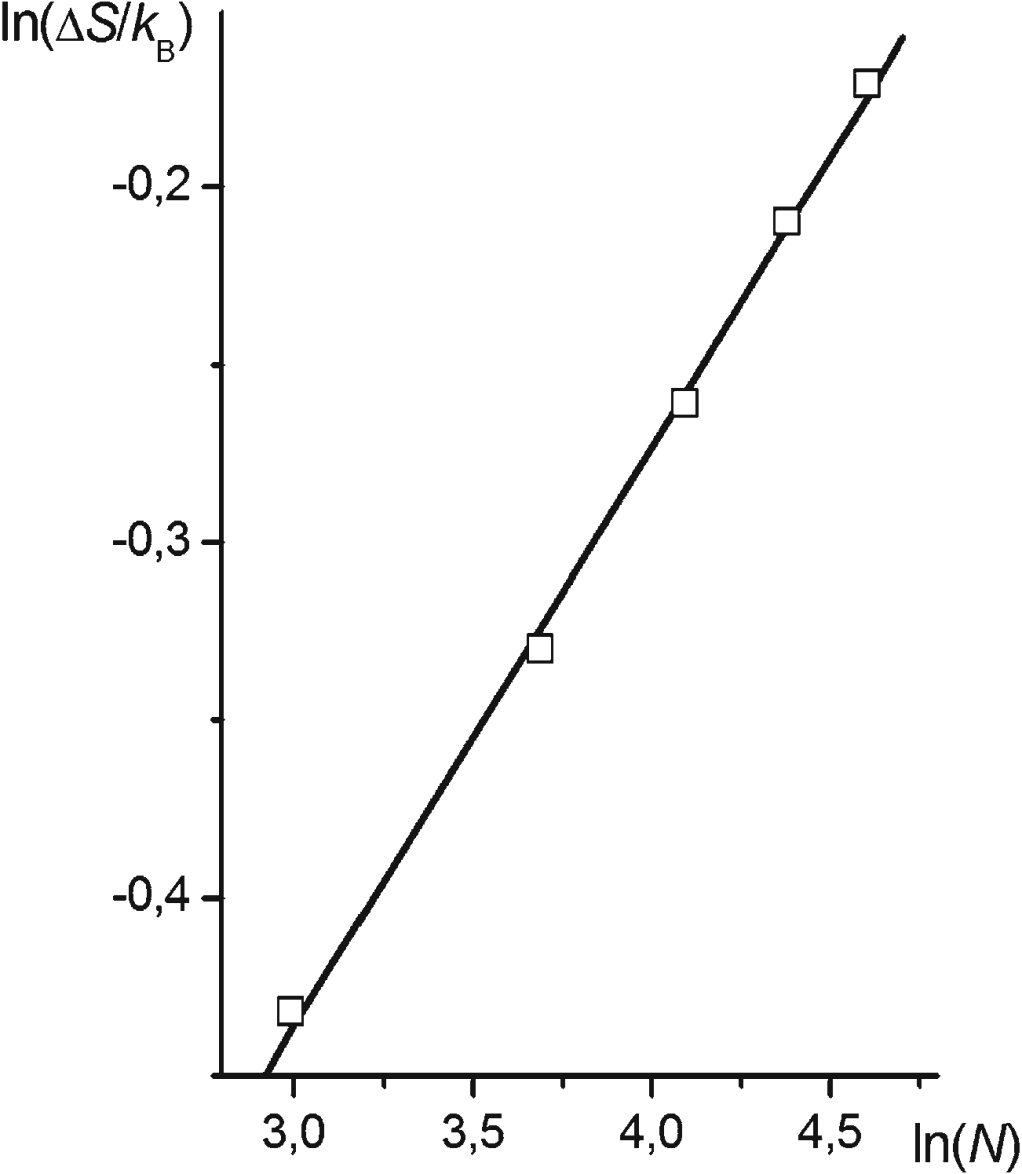
The dependence of the entropy of chain anchoring on the number of segments

Numerical calculations of the entropy of chain anchoring vs. the number of segments, based on Eqs. 13–16, produce a straight line dependence in log-log coordinates (Fig. 12). Fitting the parameters of the linear equation

17 $$ \ln \left( {\frac{{\Delta S}}{{{k_{\text{B}}}}}} \right) = \zeta \ln (N) + A $$

gives *ζ* equal to 0.162.

The value of *ζ* obtained from Eq. 17 agrees quite well with that found by simulating chains attached at *z*_mean_ and *z*_random_ on both Bm and fBm surfaces (in the studied range of Hurst parameter values), which equals to about 0.17. This agreement allows one to conclude that the simple model based on the analysis of the chain segment distribution is adequate enough to allow the prediction of some properties of a system composed of a linear macromolecule end-grafted to a rough surface. However, note that distributions (13) and (14) are continuous and can only approximately describe the effects that occur at the length scale in which the discrete properties of the lattice control system behavior.

The model given by Eqs. 13–17 can be simplified by eliminating the excluded volume effect [i.e.,* ρ*_E_(*r*) = 1] to give the form that describes an ideal polymer solution. This simplification gives a segment density in the coil corresponding to that obtained in the theta condition. However, it is important to bear in mind that the consistency of the linear dimensions of coils in theta solutions with those in the ideal solution results from compensating for the excluded volume effect using attractive intramolecular (segment–segment) interactions. Since both factors can influence the segment density near the rough surface in unpredictable ways, the possibility of extending the model to theta conditions requires further investigation.

## Conclusions

In this work, the effect of surface roughness on the conformational entropy of a terminally attached chain was studied using the lattice model. The reduction in the chain entropy upon its attachment to a surface is a complex function of the surface heterogeneity. We found that the magnitude of the entropy reduction depends not only on the values of parameters that characterize the surface roughness (i.e., on the fractal dimension or/and the standard deviation of the surface altitude) but also on the geometric details of the surface (e.g., on whether it is brush-like or quasi-fractal). In the latter case, the behavior of the modeled system (i.e., of the macromolecule at the rough surface) as a consequence of its lattice character depends on the range of dimensions of the surface inhomogeneities. The complicated courses of the curves describing the influence of *D*_F_ on *ζ* and *A* in the small-dimension range can be correlated to the disappearance of self-similarity when the sizes of the surface inhomogeneities decrease to the atomic level (or to the value of the lattice constant in the model).

In order to explain the simulation results, a simple analytical model was developed to predict the conformational entropy of an end-anchored chain. This model assumed that the density distribution of segments in the coil formed by the bound macromolecule can be found by superposing the segment density distribution in the unbound, unperturbed coil onto that of the free lattice sites near the interface. The results obtained using this superposition model were found to be in satisfactory agreement with those gained by simulating a chain anchored at *z* = *z*_mean_ on both self-similar and self-affine surfaces.

## Electronic supplementary material

Below is the link to the electronic supplementary material.

ESM 1 (PDF 779 kb)
