# Supplementary material for: Conformational entropy of a polymer chain grafted to rough surfaces
Source: J Mol Model. 2012 Aug 24;19(1):337–48. doi: 10.1007/s00894-012-1546-5 (PMC3536995; doi:10.1007/s00894-012-1546-5)
Supplement: Supplementary file 1 — (PDF 779 kb) [file 894_2012_1546_MOESM1_ESM.pdf]

Supplement

In this supplement some of the dependencies reported in the paper are represented in a somewhat different coordinate system, namely, the standard deviation of the altitude, σ, is taken as an argument, instead of the fractal dimension of the surface.

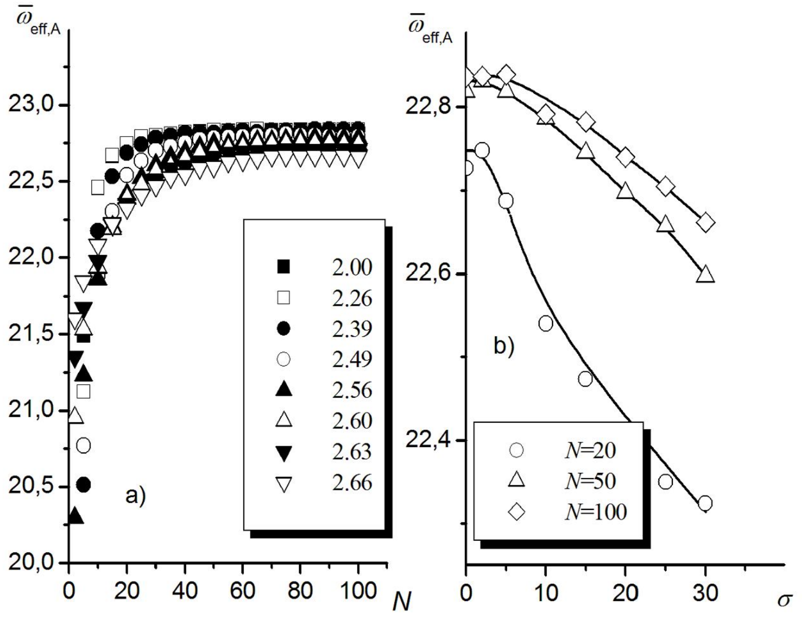

Fig. 5. Effective lattice coordination number versus a) the length of the chain anchored at uGm surfaces of different degrees of roughness – (values of D_F of the interface are indicated in the Figure) and b) the standard deviation of altitude, σ, for selected chain lengths (values of N are indicated in Figure). The chain is attached at $z=z_{rand}$.

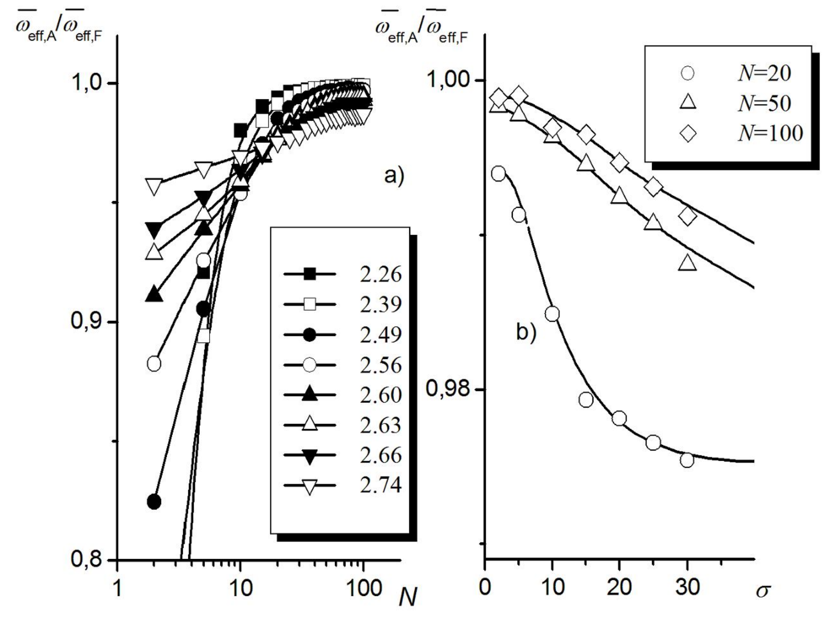

Fig. 6. The dependencies a) $\varpi_{\text{eff, A}} / \varpi_{\text{eff, F}}$ vs. N and b) $\varpi_{\text{eff, A}} / \varpi_{\text{eff, F}}$ vs. σ calculated for uGm surfaces (D_F and N values are shown in Figure, $z=z_{rand}$).

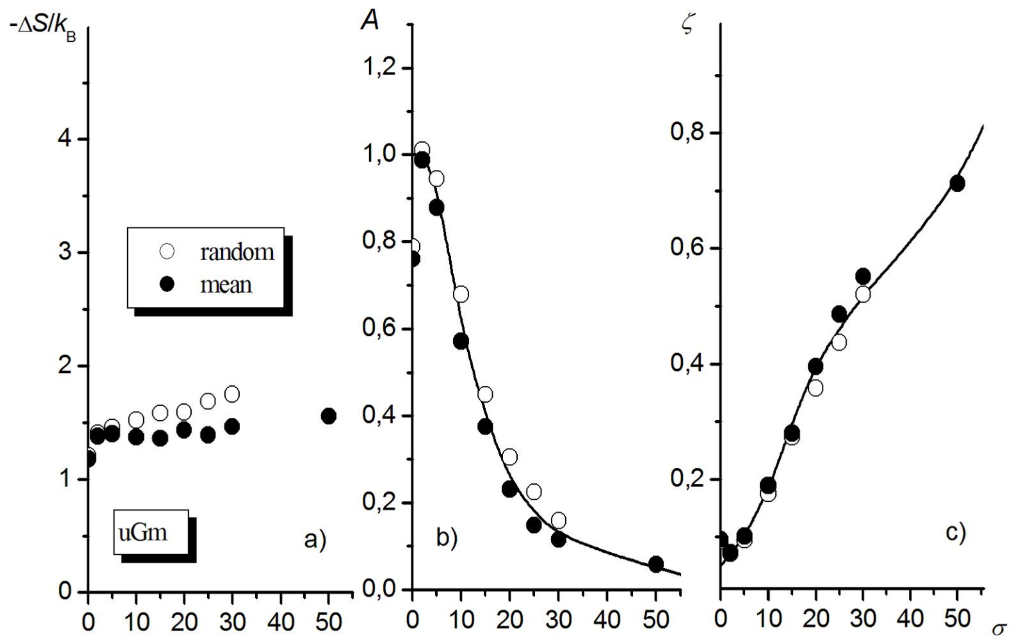

Fig. 8. Macromolecule tethered to the uGm surface. The effect of the standard deviation of altitude, σ, on ΔS (a) and coefficients A and ζ from Eq. 10 (b and c, respectively). Values obtained for different altitudes of the tethered segment are marked with different symbols as indicated in Figure.

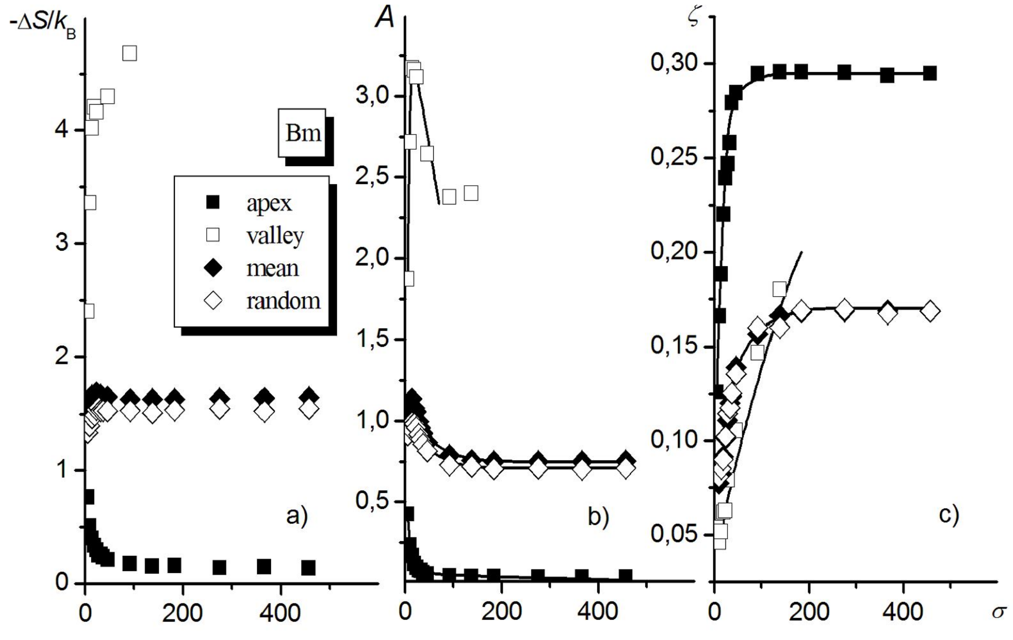

Fig. 9. Macromolecule tethered to the Bm surface. The dependencies for the same three pairs of variables as in Fig. 8 bis determined for different altitudes of the tethered segment.

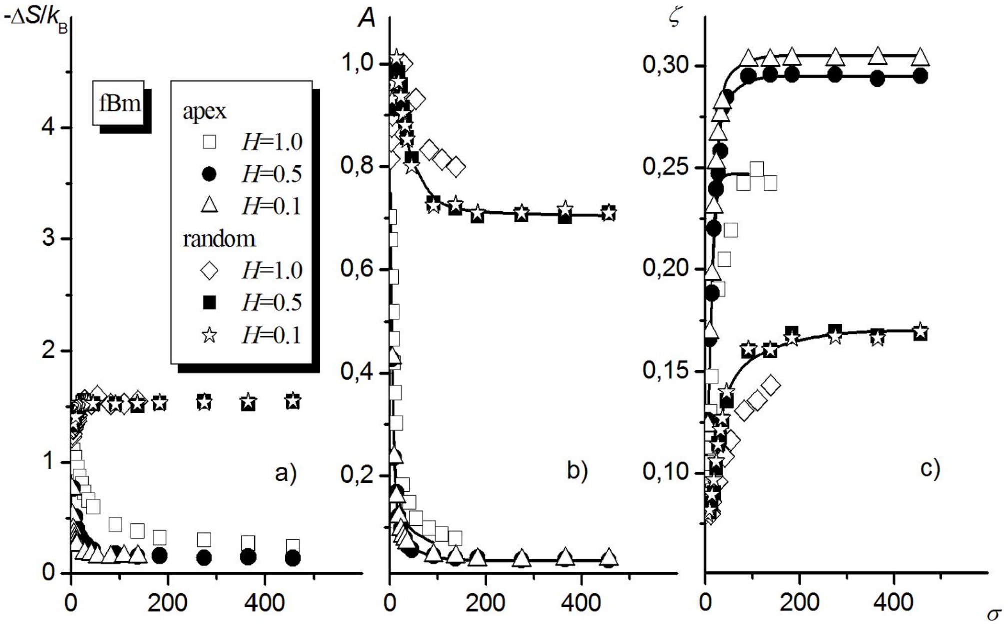

Fig. 10. Macromolecule tethered to the fBm surface. The dependencies of the entropy of chain anchoring (a) and values of the coefficient A (b) and exponent ζ (c) on the standard deviation of altitude, σ. Results obtained for different altitudes and different Hurst coefficients are marked with different symbols as indicated in Figure.
